# Assessment of pain, acceptance of illness, adjustment to life with cancer and coping strategies in breast cancer patients

**DOI:** 10.1007/s12282-015-0620-0

**Published:** 2015-06-02

**Authors:** Aleksandra Czerw, Urszula Religioni, Andrzej Deptała

**Affiliations:** Public Health Department, Medical University of Warsaw, Warsaw, Poland; Division of Cancer Prevention, Department of Oncology and Hematology CSK MSW, Medical University of Warsaw, Warsaw, Poland

**Keywords:** Breast cancer, Pain evaluation, Acceptance of illness, Quality of life

## Abstract

**Introduction:**

Breast cancer is the most common malignant neoplasm in women. Over the past 40 years, the number of patients diagnosed with breast cancer quadrupled. Breast cancer is one of the most frequent causes of death in women aged 65 and more in Poland.

**Purpose:**

The purpose of the study was to evaluate coping strategies, pain management, disease acceptance and adjustment to cancer in patients diagnosed with breast cancer and to assess the effect of socioeconomic variables on the above mentioned issues.

**Methods:**

The study included 193 patients diagnosed with breast cancer during outpatient chemotherapy (classical chemotherapy, hormone therapy, molecularly targeted therapies) at the Center of Oncology, Maria Skłodowska-Curie Institute in Warsaw. We applied the Paper and Pencil Interview (PAPI) technique. The questionnaire interview consisted of demographic questions (socioeconomic variables) and the following four psychometric tests: BPCQ (Beliefs about Pain Control Questionnaire), measuring the influence of factors affecting pain management in patients, CSQ (Coping Strategies Questionnaire), designed to evaluate pain coping strategies, AIS (Acceptance of Illness Scale) questionnaire, measuring disease acceptance, and the mini-MAC (Mental Adjustment to Cancer) scale.

**Results:**

The results of BPCQ show that breast cancer patients mostly believe that doctors control pain; the mean result for the group was 17.09 and test values were differentiated by education and professional status. The top average score in the pain coping strategies questionnaire was recorded in the positive coping self-statement subscale (mean score = 21.81), whereas the lowest, in the catastrophizing subscale (mean score = 10.60). Here, education and income proved most significant in accounting for the differences recorded. The mean score on the AIS was 28.45, and the key factor differentiating the results was income. As far as the mini-MAC is concerned, we reported the highest score in the fighting spirit subscale (23.43). The average results in the scale were slightly differentiated by socioeconomic variables.

**Conclusions:**

Breast cancer patients mostly believe that those who control pain are doctors. Amongst the strategies of coping with pain, the top average score was recorded in the positive coping self-statement subscale. We found out that the level of disease acceptance depends on respondent’s income. The higher the income, the greater the acceptance of illness.

## Background

Breast cancer is the most common malignant neoplasm in women, both in developed and developing countries [1]. Statistically 1 in 12 women suffers from breast cancer—which is 1 in every 4 cancer patients. Breast cancer is one of the most frequent causes of death in women aged 65 and more in Poland [2].

Breast cancer risk factors include age 50 or older, inherited mutation in the *BRCA1, BRCA2, ATM, PTEN, TP53,* and *CHECK2* genes, having a first-degree relative with breast cancer, early start of menstrual cycles (before age 12), late menopause (after age 55), having first child after age 30, having no children, obesity, animal fat-rich diet, routine consumption of more than two alcoholic beverages per day, long-term (more than 5 years) use of contraceptives and hormone replacement therapy, exposure to ionizing radiation, non-invasive breast cancer [ductal hyperplasia (DH), atypical ductal hyperplasia (ADH), lobular carcinoma in situ (LCIS)] and a history of the following: breast cancer, ovarian cancer, endometrial carcinoma, colorectal cancer [3].

Over the past 40 years in Poland the number of patients diagnosed with breast cancer quadrupled. In 1975, there were more than 4000 new cases. In contrast, in 2012 the same number was just below 17,000. It is estimated that in the next 15 years, the number of newly diagnosed patients will exceed 21,000 and the incidence rate will be only slightly lower than the average breast cancer incidence rate in Europe [2].

When we compare Polish statistical data with those from Europe and the rest of the world, the incidence rate in Poland remains at an average level. For instance, in Great Britain, Denmark, Finland, the United States, or Canada the number is several times higher. What is worrying, though, is that despite higher breast cancer incidence rates in the enumerated countries, the death rate due to the disease is comparable to that in Poland [4].

Cancer, and in particular breast cancer involving mastectomy, is a heavy mental burden for affected women. There are numerous assessment methods providing effective psychological support to breast cancer patients [5–7].

## Purpose

The purpose of the study was to evaluate coping strategies, pain management, disease acceptance, and adjustment to cancer in patients diagnosed with breast cancer. In addition, we also analyzed the effect of socioeconomic variables (education, professional status, income, place of residence) and chemotherapy on the above mentioned issues.

## Methods

The study included 193 patients diagnosed with breast cancer, undergoing outpatient chemotherapy (classical chemotherapy, hormone therapy, molecularly targeted therapies) at the Center of Oncology, Maria Skłodowska-Curie Institute in Warsaw in the year 2013. We applied the Paper and Pencil Interview (PAPI) technique. The questionnaire interview was composed of demographic questions (socioeconomic variables) and the following four psychometric tests:

1. Beliefs about Pain Control Questionnaire (BPCQ) designed to assess patients in pain.
2. Pain Coping Strategies Questionnaire (CSQ) used to evaluate patients suffering from pain.
3. Acceptance of Illness Scale (AIS), measuring adjustment to disease.
4. Mental Adjustment to Cancer (mini-MAC) scale, measuring the level of mental adjustment to disease.

To analyze the study findings, we applied the ANOVA and Kruskal–Wallis tests. We used the Mann–Whitney *U* test for the comparison of differences between the study groups. We treated *p* values less than 0.05 as statistically significant.

Test scores were correlated with socioeconomic characteristics of the respondents: education, professional status, place of residence, net income-per-household-member, and chemotherapy in the past year.

The study was conducted with the approval of the Bioethics Committee at the Medical University of Warsaw on 16 April 2013.

The patients were informed that the study was carried out by the Medical University of Warsaw and familiarized with the study purpose. Each study subject was informed that the results obtained would be used for research purposes only. The study included individuals who gave informed, non-written consent to participate. All individuals included in the study were adults.

## Results

### Pain Control

The statements which form the Beliefs about Pain Control Questionnaire (BPCQ) measure the power of individual beliefs regarding pain management: personally (internal factors), through the effect of doctors (powerful others), and by chance events [8].

**Fig. 1:**
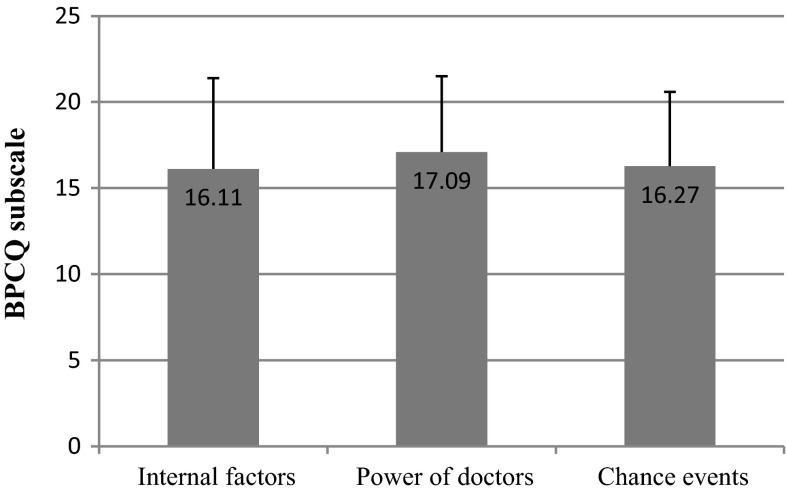
BPCQ test scores in breast cancer patients

The results of BPCQ show that breast cancer patients mostly believe that the greatest role in pain control is played by doctors, with the group mean score of 17.09 and the standard deviation of 4.40. In the case of chance events, the mean score for the patients was 16.27 and the standard deviation was 4.32. The average result for internal factors, in turn, was 16.11 and the standard deviation was 5.28 (Fig. 1).

**Fig. 2:**
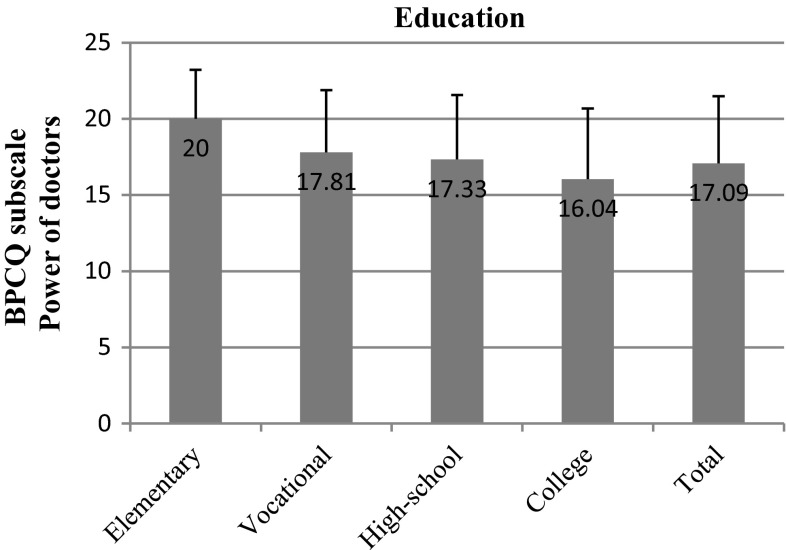
BPCQ test scores (power of doctors) in breast cancer patients vs education

**Fig. 3:**
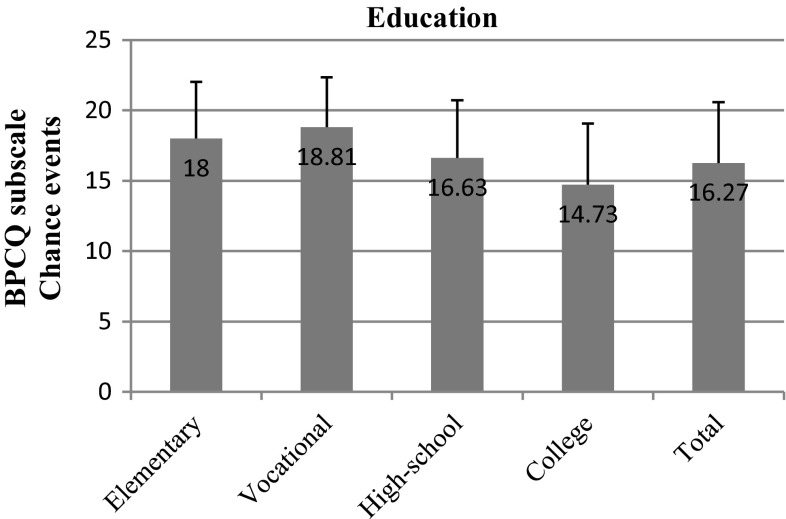
BPCQ test scores (chance events) in breast cancer patients vs education

It turned out that the most crucial factors in pain management in breast cancer patients were education and professional status. We observed that the power of doctors clearly decreased as the level of education increased (*p* = 0.014). In participants with vocational education, the mean result in this subscale was 17.81; in high-school graduates it was 17.33, whereas in respondents with a higher level of education it was only 16.04 (Fig. 2). Furthermore, we observed a negative correlation between the tendency to attribute power to chance events and an increase in the level of education (*p* = 0.000). Respondents of vocational education had a mean score of 18.81; those of high-school education, 16.63; while college graduates, only 14.73. The standard deviation in the case of patients with vocational education was 3.54, whereas in high-school and college graduates it was only 4.01 and 4.35, respectively (Fig. 3).

**Fig. 4:**
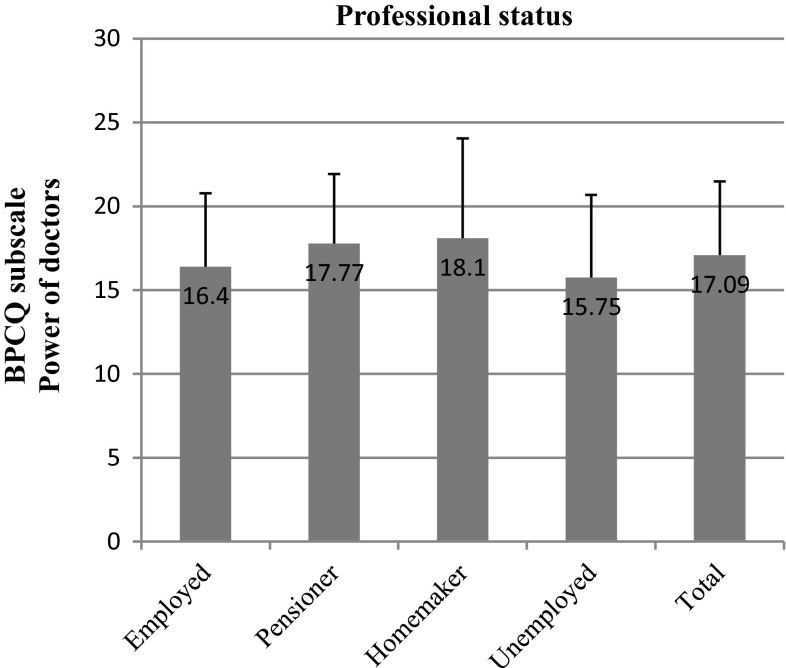
BPCQ test scores (power of doctors) in breast cancer patients vs professional status

The belief that doctors control pain was further diversified by the professional status of the respondents (*p* = 0.021). Patients who were retired attributed more power to doctors (means test score = 17.77) than did patients at a working age (mean = 16.40) (Fig. 4).

The remaining socioeconomic variables (place of residence, marital status, income) did not significantly differentiate the BPCQ scores.

### Strategies of coping with pain

The Coping Strategies Questionnaire is designed to evaluate patient strategies of coping with pain and to verify their effectiveness in pain reduction or control. Methods of coping with pain reflect six cognitive strategies and one behavioral strategy, which in turn are a part of the following three components: cognitive coping, diverting attention and undertaking replacement activities, catastrophizing and seeking hope [9, 10].

**Fig. 5:**
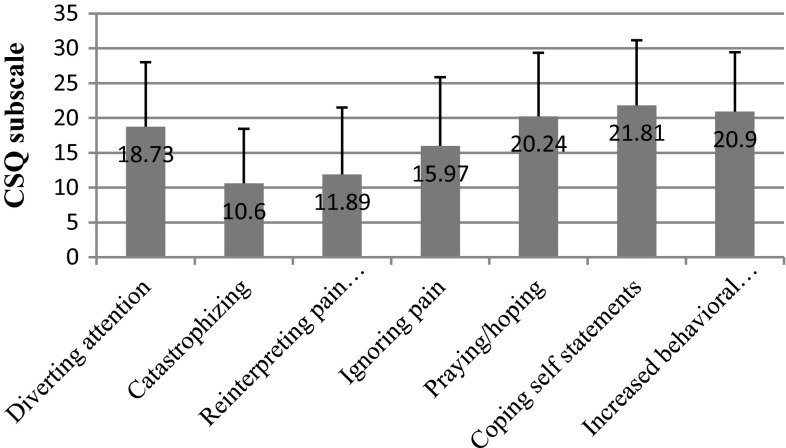
CSQ scores in breast cancer patients

We recorded the top average score for breast cancer respondents in the coping self-statement subscale (mean = 21.81), and the lowest score in the catastrophizing subscale (mean score = 10.60) (Fig. 5).

**Fig. 6:**
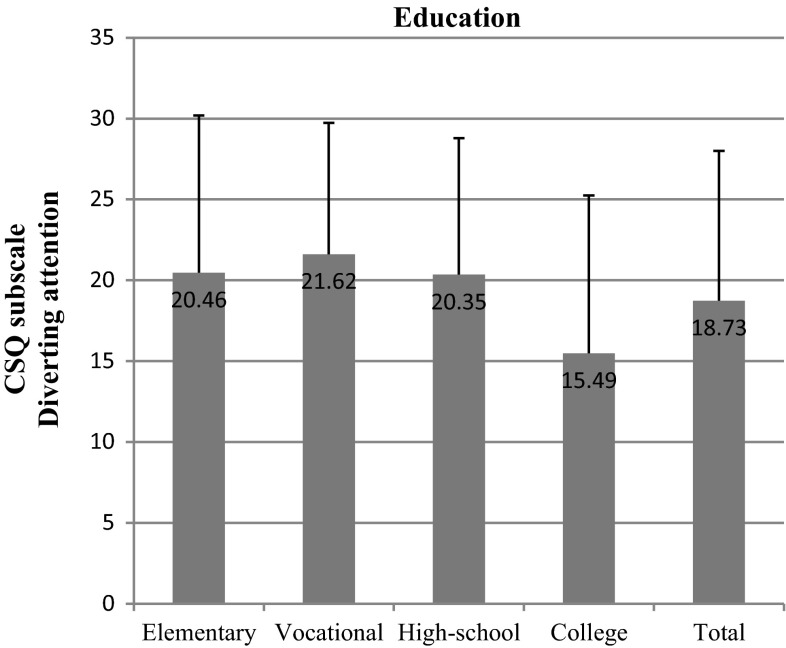
CSQ scores (diverting attention) in breast cancer patients vs education

**Fig. 7:**
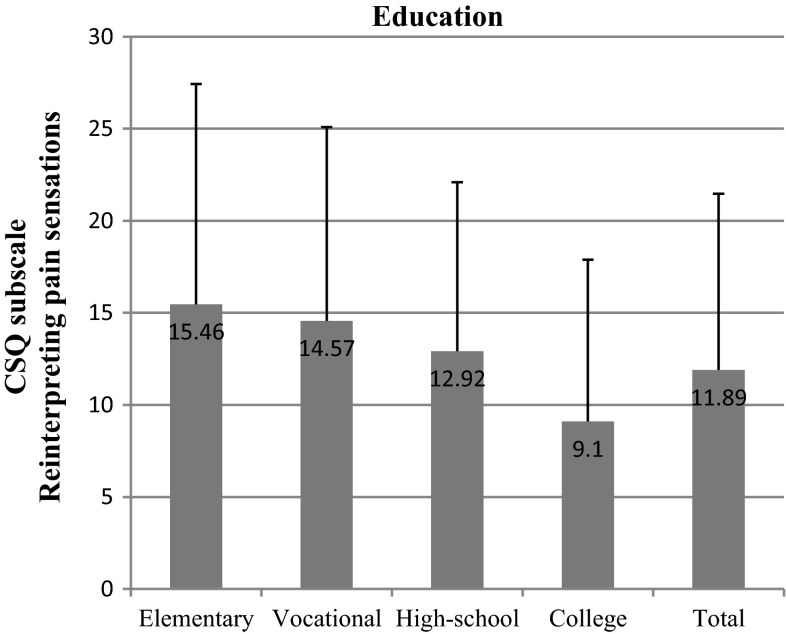
CSQ scores (reinterpreting pain sensations) in breast cancer patients vs education

**Fig. 8:**
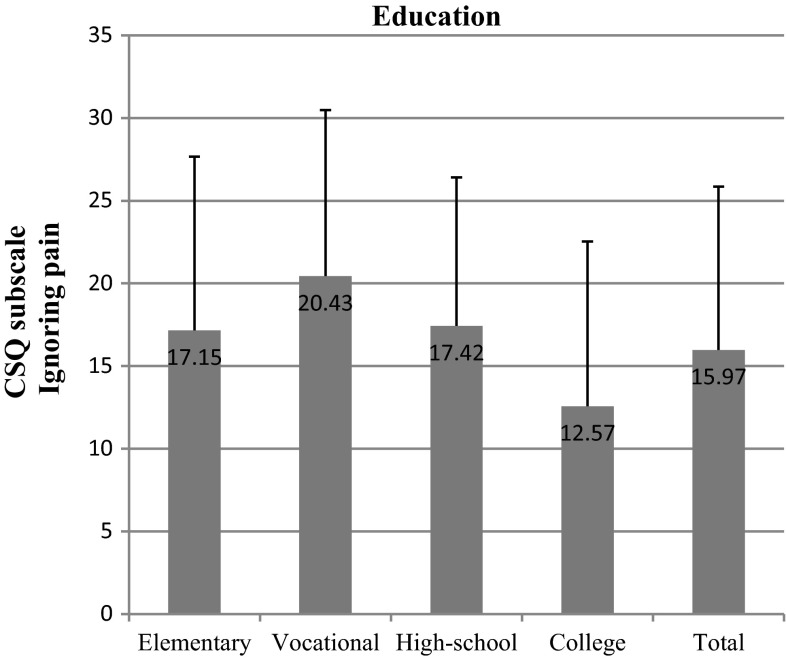
CSQ scores (ignoring pain) in breast cancer patients vs education

**Fig. 9:**
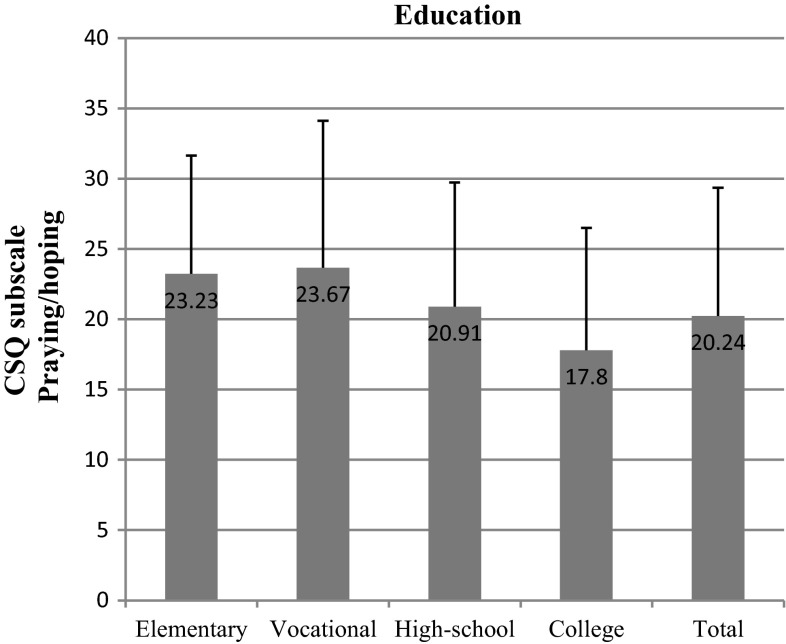
CSQ scores (praying/hoping) in breast cancer patients vs education

**Fig. 10:**
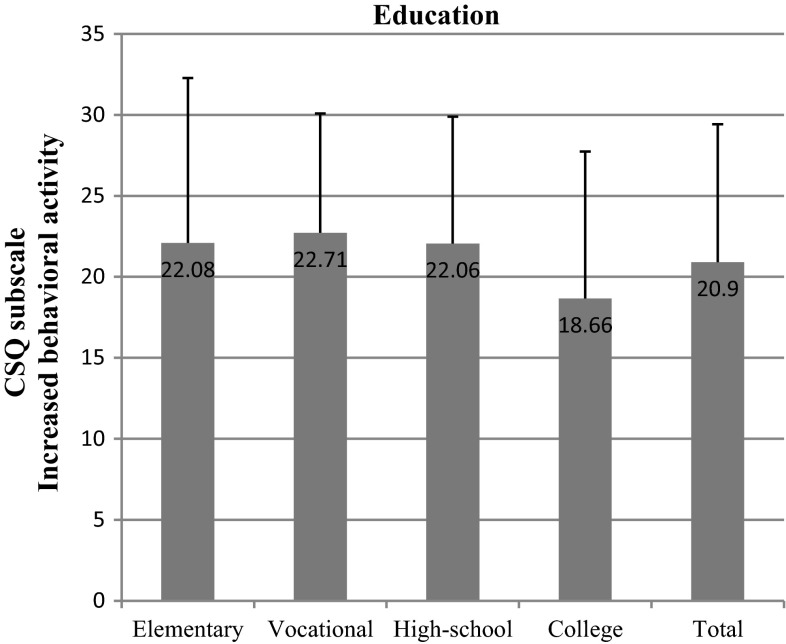
CSQ scores (increased behavioral activity) in breast cancer patients vs education

We found out that the most important socioeconomic variables that affected test scores were education and net-income-per-household-member. We observed significant differences within the “education” variable in nearly all subscales: diverting attention (*p* = 0.002) (Fig. 6), reinterpreting pain sensations (*p* = 0.006) (Fig. 7), ignoring pain (*p* = 0.001) (Fig. 8), praying/hoping (*p* = 0.041) (Fig. 9), and increased behavioral activity (*p* = 0.015) (Fig. 10). We registered a noticeable drop in the mean scores along an increase in education, but the real difference we could see only between vocational- and high-school graduates and college graduates. The groups treated individual strategies similarly and only in the reinterpreting pain sensations subscale the mean scores of respondents were lower.

Income-per-household-member differentiated the scores obtained in the following subscales: diverting attention (*p* = 0.011), reinterpreting pain sensations (*p* = 0.011), and praying/hoping (*p* = 0.001). The effect of the above strategies was inversely correlated with income. A similar correlation was observed in the case of the other subscales, but the differences recorded were not statistically significant.

The size of the place of residence and professional status of the study subjects had no effect on the strategies of coping with pain. In the first case, we recorded significant differences between individual income groups only with regard to the ignoring pain subscale (*p* = 0.042). No significant differences were registered in any of the subscales between those in employment and the pensioners.

### Disease acceptance

The Acceptance of Illness Scale (AIS), a tool for measuring disease acceptance, is composed of eight statements forming a single scale. The total score of every respondent may be between 8 and 40. The lower the score, the more intense the negative reactions and emotions related to disease and hence the lower acceptance. The higher the score, the better the adjustment to illness and the lower mental discomfort.

**Fig. 11:**
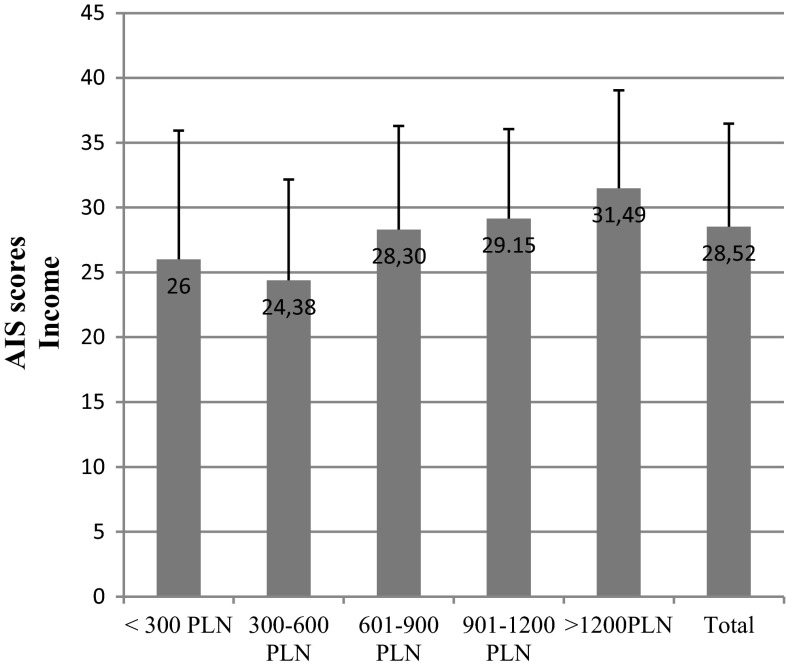
AIS scores in breast cancer patients vs net income per household member

The mean test score for breast cancer patients was 28.45 and the standard deviation was 7.98. The major economic factor that differentiated the results was income (*p* = 0.01). We could see a linear correlation between the rise in the net income-per-household-member and the AIS score. The scores ranged from 24.38 in low-income respondents to 31.49 in high-income patients (Fig. 11).

**Fig. 12:**
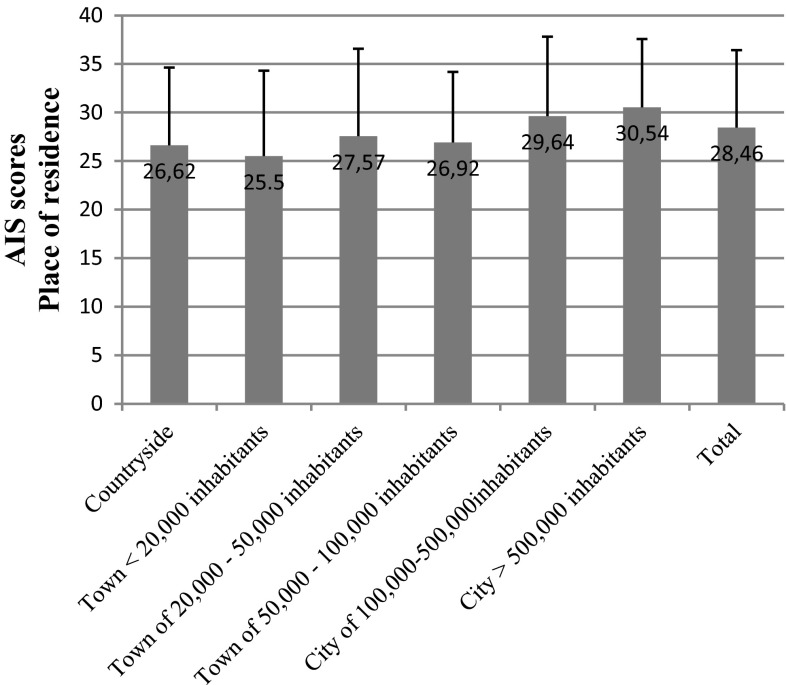
AIS scores in breast cancer patients vs place of residents

We detected a similar tendency with regard to the place of residence (*p* = 0.035). Patients living in large cities had higher scores and hence presented better adjustment to disease (Fig. 12).

AIS test scores were not differentiated by education or professional status.

### Mental adjustment to disease

The mini-Mental Adjustment to Cancer (mini-MAC) scale measures four methods of coping with disease: anxious preoccupation, fighting spirit, helplessness-hopelessness, and positive re-evaluation. While the two former methods are a part of the passive (destructive) style of coping, the latter two refer to the active (constructive) way of managing disease [11, 12].

**Fig. 13:**
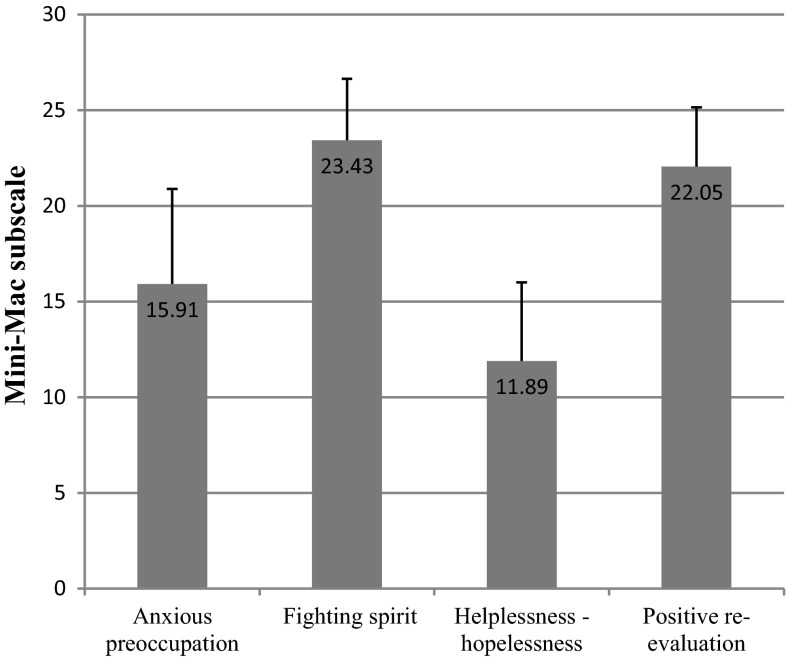
Mini-MAC test scores in breast cancer patients

Breast cancer patients had the highest mini-MAC scores in the fighting spirit (23.43) and positive re-evaluation (22.05) subscales, and the lowest in the helplessness-hopelessness subscale (11.89) (Fig. 13).

Differences between individual socioeconomic groups were minor. The only statistically significant difference we recorded was for the professional status in the positive re-evaluation subscale (*p* = 0.008).

Chemotherapy in the preceding 12 months divided the patients into two categories: respondents who were administered chemotherapy obtained higher results in the anxious preoccupation and helplessness-hopelessness subscales, as opposed to the fighting spirit and positive re-evaluation categories, where they obtained lower scores. Nevertheless, the observed differences were significant only with regard to the anxious preoccupation subscale: 17.25 in patients who were undergoing chemotherapy and 15.43 in those who were not (*p* = 0.032).

## Discussion

The International Association for the Study of Pain (ISAP) defines pain as an unpleasant experience accompanying and existing or potential tissue risk, and in some cases, also real tissue damage [13]. More than a half of cancer patients report pain [14]. However, pain therapy may be highly difficult owing to its subjectivity and the fact that not only pain intensity but also reaction to painkillers may vary from patient to patient [15].

Many studies [16] demonstrate that the locus of pain control depends on the level of disease acceptance. For instance, breast or ovarian cancer patients who demonstrated low cancer acceptance had little faith in the internal control of pain, while at the same time they attributed a great role in pain management to powerful others (doctors). Moreover, in the study group the highest scores we noted in BPCQ were in the beliefs that doctors controlled pain. The patients attributed the lowest value to internal factors. Similarly to studies with other patient groups, patients often stress that most important in pain management are doctors [17, 18].

Patients adopt various strategies in an attempt to reduce pain [19]. In our study, the patients valued coping self-statements and increased behavioral activity the most, and catastrophizing and reinterpreting pain sensations the least. Rosenstiel and Keefe, who analyzed chronically ill patients, arrived at similar conclusions [9] and so did Juczyński [20].

As already indicated in the study, the most important socioeconomic factors according to a breast cancer patient when selecting a coping strategy are education and income. The correlation between a chosen strategy and education is also demonstrated by Andruszkiewicz et al. [21], who examined a group of patients suffering from osteoarthritis of the hip. Many researchers reckon that praying and catastrophizing, the strategies indicated mostly by elementary-, vocational- and high-school graduates, worsen the general health condition and magnify the feeling of anxiety [20, 22, 23]. The World Bank data also suggest that there is a correlation between the variables recorded in our study and population’s health. According to the idea of the World Bank, health as a function of wealth and education of the society is a form of capital to be accumulated. The wealthier and more educated the society, the higher the health status of its citizens. On the other hand, a healthier society may contribute to faster economic growth of a country [24].

Breast cancer patients exhibit a relatively high level of disease acceptance (mean = 28.45) when compared to other patient groups. Juczyński [25], who conducted studied in various patient groups, concluded similarly; he demonstrated that breast and uterine cancer patients showed a much higher level of acceptance of illness (mean score = 28.13) than diabetic patients (24.81), males post myocardial infarction (22.14), males with chronic pain (18.46), and females diagnosed with migraine (24.23). Felton et al. [26], who studied chronically ill patients, and Wiraszka and Lelonek [27], who analyzed leukemia patients, also recorded lower scores (28.08 in the case of the former). Many authors indicate that higher disease acceptance leads to greater motivation to fight disease and better health management [28–30].

Breast cancer patients in our study presented an active style of coping with illness, which is an important element affecting longer survival and better quality of life [31, 32]. The study group had the highest score in the mini-MAC test in the positive re-evaluation (22.05) subscale, and the lowest in the helplessness-hopelessness one (11.89). Although our score recorded in the anxious preoccupation subscale was relatively low (15.91), that of mammary cancer patients in the study by Juczyński was 20.10 [12]. Furthermore, we demonstrated that the mean score of the constructive style of coping with disease was 45.48 and of the destructive one, 27.80, whereas Juczyński reports 40.30 and 35.80, respectively.

Kozak, who analyzed a group of females with cancers of the reproductive system, reported results that are comparable with ours. When compared to gastric, pancreatic, colorectal or prostate carcinoma, her group scored highest in the fighting spirit strategy (23.95) and lowest in the hopelessness-helplessness method [33]. Szczepańska-Gieracha et al. [34] also point out the advantage of breast cancer patients’ constructive coping strategies over other groups.

Malicka et al. draw attention to the effect of physical activity on one’s attitude to disease. On the basis of a study conducted in women post breast cancer treatment, they concluded that patients who participated in at least 5 different types of activities per week displayed higher results in the fighting spirit category. Especially important were tours and dancing, which improved mini-MAC test scores in the areas of positive re-evaluation and the constructive coping style [35]. Such correlations were further confirmed by other researchers, amongst them Lueboonthavatchai [36] and Pinto et al. [37].

What is more, numerous studies demonstrate that adopted strategies of coping with disease differ with regard to time from diagnosis and treatment stage [33, 38, 39]. For instance, in the study by Szczepańska-Gieracha et al. carried out in breast or reproductive organ cancer outpatients, the mean fighting spirit score was 24.1; the score of patients after treatment was 11.1. Similarly, the positive re-evaluation strategy results tend to decrease as the time from diagnosis increases. The helplessness and anxious preoccupation strategies remained stable regardless of the period of time after diagnosis [35].

## Conclusions

1. Breast cancer patients believe that most important in pain management are doctors, and the least important to be the internal factors.
2. Of all strategies of coping with pain, the highest mean score is recorded in the positive coping self-statements section and the lowest in the catastrophizing subscale.
3. The level of disease acceptance depends on breast cancer patients’ income. The higher the income, the more accepted the disease.
4. With regard to mental adjustment to disease, the top scores are reported in the fighting spirit and positive re-evaluation subscales.
5. The main socioeconomic variables which differentiate the scores obtained in individual tests used in our research are education and net income-per-household-member.
